# Fermentation Affects the Antioxidant Activity of Plant-Based Food Material through the Release and Production of Bioactive Components

**DOI:** 10.3390/antiox10122004

**Published:** 2021-12-16

**Authors:** Yan-Sheng Zhao, Aya Samy Eweys, Jia-Yan Zhang, Ying Zhu, Juan Bai, Osama M. Darwesh, Hai-Bo Zhang, Xiang Xiao

**Affiliations:** 1 School of Food and Biological Engineering, Jiangsu University, Zhenjiang 212013, China; zhaoys@ujs.edu.cn (Y.-S.Z.); ayasamy193@yahoo.com (A.S.E.); jiayanzhang1988@163.com (J.-Y.Z.); ying307@126.com (Y.Z.); 1000005134@ujs.edu.cn (J.B.); 2 Food Science Department, Faculty of Agriculture, Cairo University, Giza 12613, Egypt; 3 Agricultural Microbiology Department, National Research Centre, Cairo 12622, Egypt; darweshosama@yahoo.com; 4 Hubei Provincial Key Laboratory of Yeast Function, Angel Yeast Co., Ltd., Yichang 443004, China; zhanghb@angelyeast.com

**Keywords:** plant-based food, fermentation, antioxidant activity, chemical composition, phytochemicals

## Abstract

This review reports on the effects of fermentation on the chemical constituents and antioxidant activity of plant-based food materials. Fermentation involves a series of reactions that modify the chemical components of the substrate. It could be considered a tool to increase the bioactive compounds and functional properties of food plant materials. Oxidative damage is key to the progression of many human diseases, and the production of antioxidant compounds by fermentation will be helpful to reduce the risk of these diseases. Fermentation also can improve antioxidant activity given its association with increased phytochemicals, antioxidant polysaccharides, and antioxidant peptides produced by microbial hydrolysis or biotransformation. Additionally, fermentation can encourage the breakdown of plant cell walls, which helps to liberate or produce various antioxidant compounds. Overall, results indicated that fermentation in many cases contributed to enhancing antioxidants’ content and antioxidant capacity, supporting the fermentation use in the production of value-added functional food. This review provides an overview of the factors that impact the effects of fermentation on bioactive compound composition and antioxidant activity. The impacts of fermentation are summarized as a reference to its effects on food plant material.

## 1. Introduction

Today’s increased awareness of functional foods has resulted in attempts to modify available food to have higher health benefits. Plants, especially medicinal and edible plants that have high phenolic content and other bioactive compounds, can be used for the production of functional foods mainly because of the antioxidant capacity of its phytochemicals [1]. Natural antioxidants in foods are getting more and more attention due to their health and functional properties, and the expectation is that the development of natural antioxidants, such as proteins, flavonoids, and phenolic compounds inside plants, will enhance safety. For instance, phenolic compounds are naturally occurring compounds that consist of several groups, including phenols, flavonoids, tannins, and phenolic acids [2]. Much past research has focused on different methods to increase phenolic compounds in an effort to decrease or eliminate oxidative damage in the body. It has been recommended that some treatments induce levels of antioxidant activity and biologically active compounds [3]. The phenolics in food products could be affected by processing techniques, such as fermentation [4,5], enzymatic pretreatment [6], autoclaving pretreatment [7], pulsed electric fields (PEF) treatment [8], ultrasound-assisted extraction (UAE) [9], and sun-drying [10,11]. Fermentation is a biotechnological process that can improve the nutritional value and organoleptic characteristics of food by converting conjugated phenolic forms to free phenolic forms using enzymes produced by microorganisms [4]. It can also alter composition by changing protein fractions [12]. Thus, fermentation deserves attention due to its potential health benefits, such as lowering the risk of chronic diseases and cardiovascular diseases. This work provides an overview of the influence of fermentation on the antioxidant activity of different plant-based food items.

## 2. Impact of Fermentation Strain on Antioxidant Activity of Plant-Based Food Material

### 2.1. Single Strain Fermentation

Single-strain fermentation has typically been used to explore how microorganisms affect food materials. A functional and effective single strain would be more suitable for industry fermentation. Meanwhile, the properties of different strains could be screened and compared during single-strain fermentation. Hur et al. noticed that fermentation could increase the phenolic content and antioxidant activity of different plants in a manner that depended on the starting microorganisms [13]. Moreover, Ricci et al. concluded that the metabolism of phenolics depends on strain features and species [14]. For example, fermentation with *cerevisiae* had no significant effect on the inhibitory influence of 2, 2-Diphenyl-1-picrylhydrazyl (DPPH) in some cereal species, while fermentation with *L. rhamnosus* had a positive impact on the DPPH inhibitory effect in the same cereal species [15].

#### 2.1.1. Yeast Fermentation

Yeast has been one of the most widely-used microorganisms in food fermentation. The use of yeast is integral to processes such as dough fermentation and beer brewing, showing that antioxidant activity is positively correlated with yeast fermentation. For example, flavonoids, a subclass of phenolic compounds, were found to increase during the first 12 h of tea fermentation with yeast, followed by a gradual decrease. The total phenolic content increased during the first 24 h of fermentation, followed by a slight decrease after 48 h [16]. Further, yeast fermentation improved the radical scavenging activity of black tea, increasing metal chelating activities, DPPH, and ABTS (2,2′-azino-bis(3-ethylbenzothiazoline-6-sulfonate diammonium) during the 24 h of fermentation. In addition, Ooi et al. investigated the DPPH free radical scavenging activity, total flavonoid content, and total polyphenols content after fermentation of cocoa beans with *Hanseniaspora thailandica* and *Pichia kudriavzevii*. The result showed that yeast fermentation increased antioxidant potential [17]. However, Laaksonen et al. [18] found that fermentation with commercial yeast had only a minor impact on the phenolic product contents. Authors used the commercial yeast strains *Saccharomyces bayanus*, *Saccharomyces cerevisiae*, and *Torulaspora delbrueckii,* and the results showed that fermentation with *S. bayanus* produced freer hydroxycinnamic acids (phenolic compounds) than fermentation with *S. cerevisiae,* indicating higher enzyme production for breaking the ester bonds between quinic acid and hydroxycinnamic acids. In summary, according to previous research, selected yeast strains impact fermentation results by increasing antioxidant activity, flavonoids, and metal chelating activity.

There are several different yeast strains for food fermentation, such as *Saccharomyces cerevisiae*, *Saccharomyces bayanus*, *Torulaspora delbrueckii*, *Hanseniaspora thailandica*, and *Pichia kudriavzevii* [19]. Kim et al. demonstrated that antioxidant activity has a positive correlation with the yeast fermentation time [20]. Polyphenols are the major natural antioxidants in food, as illustrated by Verni et al. [21]. Additionally, Reis et al. showed that fermentation of goji berries with *Saccharomyces cerevisiae* increased the total phenolic content [22]. Anson et al. fermented wheat bran with *Saccharomyces cerevisiae* and noticed an increase of p-coumaric, sinapic, and ferulic acids content three-fold higher than unfermented wheat bran [23].

#### 2.1.2. Fungal Fermentation

Similar to yeasts, fungi have been frequently used in the food, condiment, and brewing industry, as a starter to convert the starch in raw materials into fermentable sugars. Ajila et al. [24] found that carbohydrate-degrading enzymes produced by fungi during fermentation convert conjugated phenolic compounds to free form, which improves their ability to work as good antioxidants. They showed that an increase in phenolic amounts during fermentation might be attributed to the fungi β-glucosidases which can hydrolyze β-glucosidic linkages, then mobilize phenolic compounds to react with the Folin–Ciocalteau reagent. Accordingly, *Aspergillus niger* is the most used microorganism in solid-state fermentation because of its ability to produce a large number of enzymes [25]. Hifney et al., who used various fungal species to ferment the seaweed *Cystoseira trinodis* before extracting alginate and fucoidan, showed that all the fungal species chosen produced alginate lyase and fucoidanase, decreasing the molecular weight of alginate and fucoidan. This process led to the enhancement of the antioxidants’ ferric-reducing power (FRAP), hydroxyl radical scavenging activity (HRSA), and total antioxidant capacity (TAC) of fucoidan and alginate [26]. However, some studies indicated that not all fermentations play a positive role in antioxidation activity. For example, it was found that *Aspergillus carbonarius* fermentation increased the antioxidant activity of *Cabernet Sauvignon* grapes but did not affect the antioxidant activity of *Moscato Italico* grapes, demonstrating that fruit variety has an impact on fermentation results [27]. Additionally, *Monascus purpureus* fermentation of defatted soybean flour increased isoflavone content but caused almost no change in total phenolic content or antioxidant activity. Thus, the increase in isoflavone content could be attributed to the selective action of β-glucosidase, which may result in polyphenol aglycones having a lower capacity for reducing the Folin–Ciocalteu reagent, or alternatively, to the synthesis of β-glucosidases having low activity on other soybean flour phenolic glucosides [28]. On the other hand, the results from Lee et al. showed that the total isoflavone content of diseased soybeans decreased significantly due to *Phomopsis longicolla* and *Cercospora kikuchii* fungi [29], which suggests that the effects of different fungi on antioxidant compounds in plants may be selective.

#### 2.1.3. Bacterial Fermentation

It is well known that lactic acid bacteria have been applied in fermented dairy, fermented vegetables, and fermented meat products in the food industry. The potential of lactic acid bacteria for enhancing the antioxidant activity of plant materials may be due to a decrease in pH and the enzymolysis effect during fermentation. Tkace et al. studied the effect of fermentation by using the *Oenococcus oeni*, *Lactobacillus plantarum*, and *Lactobacillus plantarum* subsp. *argentoratensis* strains on 100% Sea buckthorn (*Hippophaë rhamnoides* L.) berries mixed with apple (1:1) juices [30]. It was found that *Lactobacillus plantarum* strains were effective in increasing flavonols and enhancing the antioxidant activity of sea buckthorn–apple juices. Additionally, fermentation of quinoa flour by lactic acid bacteria achieved higher antioxidant activity than spontaneous fermentation, especially with *Lactobacillus plantarum* T0A10, through proteolysis of native quinoa proteins during fermentation [31]. Additionally, fermentation by *Lactobacillus casei* increased the isoflavone aglycone and phenolic content of whole soybeans [32]. All the results above indicate that bacterial fermentation plays an important role in the improvement of antioxidant activity.

### 2.2. Mixed Strain Fermentation

Some past research has suggested that fermentation using a complex of strains was better than a single strain [15]. It was found that mixed cultures enhanced the growth of *L. plantarum* strains, which may relate to the production of nutrients, such as vitamins, by other strains [33]. The pure and mixed cultures of *L. plantarum* LP3, *L. plantarum* AF1, and *L. plantarum* subsp. *plantarum* PTCC 1896 were used to ferment bergamot juice. Mixed ternary cultures had the highest radical-scavenging effect and DPPH scavenging ability. Wang et al. fermented guava leaf tea with *Monascus anka* and *Saccharomyces cerevisiae* and then hydrolyzed the mixture with complex enzymes. Fermentation resulted in an increase in the reducing power of soluble phenolics, an increase in antioxidant activity, and inhibitory efficacy towards α-glucosidase, and the hydrolyzing enzymes increased the positive effect of fermentation [34]. They reported that antioxidant activity increased due to an increase in the solubility of phenolic compounds in the guava leaf tea extract and due to the production of more phenolic compounds with higher bioactivities, such as kaempferol and quercetin.

## 3. Impact of Fermentation on Antioxidant Activity of Various Plant-Based Food Materials

### 3.1. Cereals

Cereals exhibit good antioxidant capacity due to the phenolic compounds distributed in the outer layer of the grain, and since most phenolics exist in the bound state [35]. Fermented cereal samples (including buckwheat, wheat germ, barley, and rye) with *L. rhamnosus* had higher antioxidant activity than those fermented with *S. cerevisiae* [15]. This indicated that fermentation with *S. cerevisiae* did not significantly influence lipid peroxidation inhibition in cereals, while fermentation with *L. rhamnosus* did have such influence. Therefore, the increase in antioxidant activity depended on the selected microorganism species. Additionally, fermentation of rice bran with lactic acid bacteria increased antioxidant activity due to an increase of free and soluble conjugated phenolic compounds and the bioavailability of free hydroxyl groups [36]. Fermenting *Radix Puerariae* combined with red yeast rice led to an increase in total phenolic content and pigment intensity and to enhanced antioxidant activity [37]. By contrast, fermentation with *L. rhamnosus* and *S. cerevisiae* did not show any significant influence on the ferric-reducing antioxidant power of buckwheat, wheat germ, barley, and rye [15]. Zhao et al. fermented barley with *Lactobacillus plantarum* for one day, and it was found that saccharides, amino acids, nucleosides, and some organic acids decreased, while lipids and bioactive molecules in barley were released and metabolites were accumulated during fermentation. Moreover, characteristic components in fermented barley were revealed, including functional molecules such as indole-3-lactic acid, phenyllactic acid, homovanillic acid, cafestol, among others. [38].

### 3.2. Legumes

The functional properties of legumes and their products have been the focus of research in recent years, and their antioxidant activity has received increased attention. In this regard, Limón et al. concluded that the fermentation technology encouraged obtaining bioactive compounds from kidney beans, which led to their useful utilization in added-value functional foods [39]. Dueñas et al. reported that *Lactobacillus plantarum* for fermentation of cowpeas could enhance the antioxidant activity [40]. Starzyńska–Janiszewska et al. suggested that fermentation of legumes decreases their allergenicity and lowes the non-nutritional factors [41], and Crujeiras et al. indicated that fermented legumes exhibited protective effects against cardiovascular disease [42]. Xiao et al. found that the solid-state fermentation of chickpeas with *Cordyceps militaris* SN-18 possessed higher total phenolic content and antioxidant activity compared with unfermented samples [43]. During fermentation, phenolic compounds such as chlorogenic acid, shikimic acid, biochanin A, daidzein, and rutin. They used 80% methanol, 80% ethanol, and water for extraction and demonstrated that methanolic extracts exhibited the highest scavenging DPPH radical ability compared with other solvent extracts. Thus, solvent selection may lead to variation in DPPH radical scavenging ability. Therefore, a strong correlation has been found between specific phenolic compounds and the antioxidant activity of some fermented legumes [39]. Marazza et al. reported that the isoflavone aglycones produced by fermentation are more active antiradical compounds [44]. In addition, higher reducing power was obtained from soybean fermented with *Streptomyces* sp., *Acetobacter* sp., *Saccharomyces* sp., and *Lactobacillus* sp., which might be related to the hydrogen-donating ability of the contained reductones [45,46].

### 3.3. Vegetables

Vegetables are considered to be rich in antioxidants. Sawicki et al. investigated the effect of boiling and spontaneous fermentation on the betalains of red beetroot. It was found that matrix softening and microbial activity increased the release of betalains during fermentation [47]. Firstly, an increase in betalains content by 99% within the first seven days of the fermentation process was observed, followed by a decrease by 17% during days 8–12. Afterwards, the fermentation process of the inner tissue of red beet slices could not overcome the degradation processes. Degradation continued during the next or 13th day of fermentation, and finally, the degradation processes began to prevail in the fermented juice. The authors reported that 14-day fermented red beets had the lowest value in antioxidant activity. Although prior research has shown that storage affects the antioxidant activity of fermented products, the opposite was observed by Wiczkowski et al., who studied the effect of fermentation on the antioxidant capacity of red cabbage. Lower antioxidant capacity was found in fermented red cabbage than fresh red cabbage due to decreased anthocyanin content, contributing to antioxidant capacity [48]. However, they noted that fermented red cabbage is still rich in anthocyanin content. Olennikov et al. pointed that novel melanoidin is an essential antioxidant found in the fermented leaves of willowherb with a content of 145.31 ± 4.35 mg per gram dry plant weight, and it is worth further investigation as potential food and medical antioxidant [49].

### 3.4. Fruits

Fruits are a great source of natural antioxidants in the human diet. Dulf et al. investigated the effect of solid-state fermentation (SSF) by *Rhizopus oligosporus* and *Aspergillus niger* on the antioxidant activity of plum pomaces and noticed greater free-radical scavenging activities leading to an increase in the antioxidant activity of plum pomaces [25]., Dachery et al. also evaluated the effect of *Aspergillus carbonarius* on the antioxidant activity of *Cabernet Sauvignon* (CS, red) grapes and *Moscato Italico* (MI, white) grapes. It was found that an increase in antioxidant activity for red grapes, but a decrease in antioxidant activity for white grapes [27]. However, Jiménez-López et al. noticed only a slight increase in antioxidant activity during the fermentation of caper berries (*Capparis spinosa* L.) [1]. They mentioned that glucosinolate glucocapparin was fully degraded after the fermentation process of berries, producing methyl isothiocyanate as the main degradation product. They also observed that most phenolic compounds remained unaltered during the fermentation of caper berries except in the case of hydrolysis of quercetin glycosides and degradation of glucosinolates, which was probably due to pH changes during the fermentation process. Besides this, a decrease in epicatechin concentration was also observed.

## 4. Changes of Antioxidant Constituents in Plant-Based Food Material during Fermentation

### 4.1. Phytochemicals

Microorganisms can modify antioxidant constituents during the fermentation process. Fermentation can lead to the structural breakdown of plant cell walls, leading to the synthesis and liberation of various bioactive compounds, as shown in Table 1.

#### 4.1.1. Phenolic Compounds

Phenolic compounds are important phytochemicals found widely in plants, and they are well-known antioxidants because of the high reactivity of hydroxyl substitution and their ability to scavenge free radicals. Đorđević et al. used the yeast *Saccharomyces cerevisiae* and bacteria *Lactobacillus rhamnosus* to ferment four kinds of cereal, including wheat germ, buckwheat, rye, and barley. It was found that total phenolic content (TPC) was enhanced after fermentation. Moreover, rye had the lowest total phenolic content with 13.3 mg of gallic acid equivalents (GAE) per gram of dried extract (d.e.) in the unfermented sample, 16.2 mg GAE/g d.e. in *S. cerevisiae*-fermented extract, and 18.4 mg GAE/g d.e. in *L. rhamnosus*-fermented extract [15]. Chung et al. showed that the total phenolic contents in 12-h and 24-h fermented ginseng extracts with *Lactococcus lactis* KC24 increased by 6.1% and 4.1%, respectively, and were greater than in nonfermented extracts [54]. Moreover, Antognoni et al. used different lactic acid bacteria strains to ferment wheat (*Triticum aestivum L.*) and detected ferulic, p-coumaric, cinnamic, caffeic, sinapic, p-hydroxybenzoic, and gallic acids in the fermented dough [50]. Liu et al. also detected 12 phenolic compounds after fermentation of rice bran: ferulic acid, protocatechuic acid, isoferulic acid, gallic acid, caffeic acid, chlorogenic acid, coumaric acid, syringic acid, kaempferol, vanillic acid, catechin, and (−)-epicatechin [36]. Ricci et al. noticed that fermentation was able to increase flavanol glycosides and anthocyanins, produce phenyllactic acids, and modify hydroxycinnamic acids [14]. It was also found that catechol and dihydrocaffeic acid were produced during the fermentation of elderberry juice, but protocatechuic acid and caffeic acid were consumed by lactic acid bacteria.

The fermented elderberry juice was characterized by flavonols, flavonol glycosides, anthocyanins, hydroxybenzoic acid, phenyllactic acids, and hydroxycinnamic acids [14]. The increase in hydroxycinnamic acids could be attributed to the increase of 5-O-caffeoylquinic acid in *Lactobacillus plantarum* 285, *Lactobacillus rhamnosus* 1019, 1473, 2360, and *Lactobacillus casei* 2107, which was used to ferment the juice [14]. Phenyllactic acids can derive from amino acid metabolism, such as converting phenylalanine to phenylpyruvic acid by a transamination reaction, followed by metabolizing into phenyllactic acid by hydroxyl acid dehydrogenase [14]. Additionally, solid-state fermentation of kidney beans exhibited high soluble phenolic compound content and antioxidant activity due to the production of β-glucosidases by *Bacillus subtilis* [39]. Liu et al. noted that antioxidant activity increased due to a release of antioxidative phenolics and an increased bioavailability of free hydroxyl groups during fermentation [36]. However, Dulf et al. reported a slight decrease in the free phenolic content of plum by-products after fermentation, perhaps attributable to oxidative enzymes that polymerize released phenolics [25]. Further, most phenolic compounds remained without alteration during the fermentation of caper berries (*Capparis spinosa* L.), although there were some changes, such as a decrease in epicatechin concentration and the hydrolysis of quercetin glycosides, which may be due to pH changes during the fermentation process [1]. Even though glucosinolates were fully degraded upon fermentation, antioxidant activity slightly increased upon the fermentation process.

#### 4.1.2. Carotenoids

Carotenoids are vital compounds because of their antioxidant mechanisms [55]. Fermentation can increase the release of carotenoids [55]. For example, lactic acid fermentation can preserve β-carotene more than other processing techniques, such as deep-frying, blanching, and drying [56]. In addition, *Lactobacillus plantarum* (29DAN, 83DAN) was able to increase the carotenoid content in wheat dough due to the higher solubility of these antioxidant compounds [50]. The effect of fermentation on carotenoid concentration depends on the plant material, the carotenoid involved, the fermentation conditions, and the enzyme activity of strains because some enzymes favour carotenoid extractions [55].

#### 4.1.3. Phytosterols

Phytosterols are naturally occurring potential antioxidants that contribute to the antioxidant capacity of soy germ [57]. Stigmasterol, D-7-avenasterol, campesterol, β-sitosterol, and 5-avenasterol are examples of phytosterols [57,58]. Adeyeye compared the changes of phytosterol content in African locust bean seeds by fermentation, and the result showed a higher value in the fermented samples than the unfermented samples [58]. However, Hubert et al. used lactic acid bacteria to ferment soy germ and studied the effect of fermentation on its phytochemical composition. A reduction in phytosterol content in soy germ was observed upon the fermentation for 48 h. This could be attributed to dehydration or oxidation that occurs in an oxygen environment when substrates are exposed to heat or ultraviolet light. Additionally, it was found that phytosterols were poorly active in scavenging the superoxide anion radical in soy germ. However, it was noted that the relative distribution of the individual phytosterols remained constant during all the fermentation stages. A 6-h incubation period is the best time to obtain conserved amounts of phytosterols in fermented soy germ, perhaps due to the low pH value. Thus, controlling incubation time can help preserve the phytosterol levels [57].

#### 4.1.4. Saponins

Saponins are chemical compounds composed of a triterpenoid or steroid sapogenin nucleus with one or more carbohydrate branches [59]. The triterpenoid saponins could be classified into major groups A and B, each containing a glycone fraction attached to one or more oligosaccharide chains [57]. Saponins are natural glycosides that contribute to the antioxidant capacity of soy germ [57]. However, the soyasaponins were poorly active in scavenging the superoxide anion radical $O_{2}^{-}$. It was found that considerable changes in the conjugation profile of soyasaponins during the fermentation of soy germ by lactic acid bacteria. Moreover, it was reported that during the incubation period, the conjugation profile of soyasaponins B was modified, and 2,3-dihydro-2,5-dihydroxy-6-methyl-4H-pyran-4-one (DDMP)-conjugated saponin significantly decreased after the fermentation of soy germ with lactic acid bacteria for 10 h. This could be attributed to the loss of their terminal sugar through enzymatic hydrolysis, followed by conversion into other unidentified structures. Hubert et al. discussed that (DDMP)-conjugated soyasaponins were converted into their DDMP-moieties with the release of maltol molecules during fermentation. After a 10-h to 48-h incubation, soyasaponins B remained unchanged [57].

### 4.2. Antioxidant Peptides

Antioxidant peptides are the peptides with the function of inhibiting peroxidation and scavenging free radicals. Generally, the antioxidant activity of peptides is related to the molecular weight, amino acid sequence, and amino acid side chain group. It was reported that the addition of purified peptides to goat milk powder increased antioxidant capacities [52]. Rapeseed and bitter bean peptides exhibited high scavenging free radical activities but low ferrous ion-chelating activity [60,61]. Additionally, peptides that chelate metal ions could be used as an indirect antioxidant agent [62]. Concerning the fermentation process, Muhialdin et al. noticed that the fermentation process of bitter beans produced higher peptides content with lower molecular weight than the boiling process. Furthermore, they concluded that the increased antioxidant activity in *Lactobacillus fermentum* ATCC9338 fermented bitter beans was due to an increase in low-molecular-weight peptides [60]. Additionally, fermentation can decrease glucosinolate content, increasing the nutritional value of the hydrolyzed peptide products, while the fermentation of different food items increases peptides via protein hydrolysis through microbial proteolytic enzymes [62]. Chen et al. mentioned that the release of antioxidant peptides was affected by selected lactic acid bacteria species. Three antioxidative peptides were obtained after fermentation of goat milk with *Lactobacillus plantarum* 60 [52]. They explained that the radical scavenging activity of the antioxidant peptides depends on certain proteolytic enzymes inside bacterial strains rather than the high proteolytic state of fermented products.

### 4.3. Antioxidant Polysaccharides

Polysaccharides are important compounds that exhibit many biological activities, such as antioxidants related to the structure of polysaccharides and their molecular mass [36]. It was reported that fermentation is a good way to improve the antioxidant activities of *Auricularia auricula* polysaccharides [61]. Fermentation degraded the pyran type polysaccharide to furan type polysaccharide with improved antioxidant activity. In addition, Liu et al. noticed that the antioxidant activity of rice bran polysaccharides increased by fermentation with the *Grifola frondosa* fungus [36]. After nine days of fermentation, it was noticed that a decrease in molecular weight of the polysaccharides from the range of approximately 10^3^–10^4^ Da to the range of about 10^2^ and 10^3^ Da, with a 2.9% increase between 10^3^ and 10^4^ Da. The changes in molecular weight distribution may be due to extracellular enzymes secreted from *Grifola frondose* [36]. In other research, Liu et al. prepared a polysaccharide from *Inonotus hispidus* by fermentation and isolated two types of polysaccharide fractions and studied the characteristics of the fractions that had the higher antioxidant activity [63]. They described how fungal polysaccharides produced by solid-state fermentation were characterized by a shorter production cycle and high conductivity to biological circulation. They also showed that the polysaccharides produced by fermentation had higher antioxidant activity than unfermented polysaccharides from the fruiting body of *Inonotus hispidus*. Fermented polysaccharides reduced H_2_O_2_-induced cellular damage and improved the cell survival rate.

### 4.4. Conversion Effect of Fermentation on Antioxidant Components

As already mentioned, the antioxidant peptides and polysaccharides could be degraded or synthesized by microorganisms, usually accompanied by the conversion from large to smaller molecules. For the phytochemicals, there are not only changes in content but also to their metabolism and other biotransformation effect during fermentation. Cho et al. investigated phytochemical contents in soybean paste fermented with *Bacillus subtilis* CS90. It was found that the levels of isoflavone aglycones, flavanols, and gallic acid increased, while the β-glucosidase and esterase activities, isoflavone glycosides and flavanol gallates contents decreased [64]. Koistinen et al. processed rye bran with the combination of enzymatic processing and yeast fermentation. Different from sourdough fermentation, a significant increase of free phenolic acids in bran was observed, some of the hexose moieties were released from benzoxazinoids, and alkylresorcinols experienced moderate degradation [65]. It indicated that the conversion of phytochemicals was variance attributed to the bioprocessing type and conditions. Sheih et al. considered that the novel antioxidants could be produced from the soy germ materials fermented with *A. niger* M46. Moreover, the fermentation altered the composition of isoflavones, and the isoflavones were further converted into other microbial-induced metabolites [66]. Rodríguez et al. pointed that *L. plantarum* was able to degrade some food phenolic compounds and metabolize some of the hydroxycinnamic and hydroxybenzoic acids [67,68]. Thus, further studies are needed to investigate the fermentation characteristics of strains, the role of enzymes, and their relationship with antioxidants’ release, synthesis, and metabolism.

## 5. Effects of Various Factors on Changes to Antioxidant Components and Activities during Fermentation

### 5.1. Effect of Enzyme on Fermentation Results

An enzyme is one of the key factors to promote the release and production of bioactive components in the fermentation process of microbial growth and metabolism. There are some enzymes, such as cellulase, amylase, esterase, tannase, and glucosidase, that can break down plant cell walls, liberating or producing bioactive compounds such as flavonoids, phenolic acids, etc. [13]. *Aspergillus niger* can produce more than 19 types of enzymes, including cellulase and pectinase. As reported, increases in phenolic content in the early stages of solid-state fermentation were attributable to glycosidases and lignin-degrading enzymes, which can help release cell wall-bound phenolic compounds. However, after nine days of fermentation, a slight decrease in free phenolics of plum by-products was noticed. This may be attributed to oxidative enzymes that polymerize released phenolics [25]. Hur et al. also reported that grain constituents exposed to bacterial and endogenous enzymes caused the solubility of some components and the production of new nutritionally active molecules [13]. Phospho-β-glucosidases plays a significant role during the fermentation of plant-based foods related to the release of phenolic compounds. Acin-Albiac et al. characterized the metabolism of *Lactiplantibacillus plantarum* and *Leuconostoc pseudomesenteroides* during their fermentation with brewers’ spent grain and noted an increased metabolic activity for gentiobiose, cellobiose and β-glucoside conjugates of phenolic compounds during fermentation [69]. In addition, de Araújo et al. investigated the hydrolytic potential of the enzymes of filamentous fungi isolated from the natural cocoa fermentation. Nineteen different species of fungi were isolated, and most of them exhibited the ability to secrete enzymes, including amylase, pectinase, cellulase, and xylanase [70].

Therefore, the addition of hydrolases helped convert bound bioactive compounds into a free state, whether exogenous or endogenous. Hydrolysis can enhance the liberation of phytochemicals in food items. Solid-state fermentation was employed by Costa et al. to produce feruloyl esterase and xylanase, and the addition of fermented wheat bran and brewer’s spent grain led to an increase in soluble ferulic acid of 159% and 198% in the bioprocessing bread, respectively [71]. Moreover, Bei et al. pointed that additional time added to cellulase resulted in significant variance in phenolic content during the solid-state fermentation of oats, but there was no significant difference if cellulase was added during inoculation [51]. In summary, the addition of specific enzymes could enhance the effect of fermentation by increasing the content of bioactive substances.

### 5.2. Effect of Fermentation Conditions on Fermentation Results

As mentioned, fermentation does not always play a positive role in the release and production of bioactive components. In addition to the selection of strains, fermentation conditions are significant factors in this process. Hur et al. reported that changes in temperature during the fermentation of plant-based foods influence the resulting antioxidant activity, including, for instance, those of phenolic compounds [13]. Optimum temperature led to enhanced microbial growth and enzymatic activity that resulted in improved fermentation. Extremely low or high temperatures negatively affected antioxidant activity during fermentation. Yao et al. evaluated the antioxidant activities of *Eurotium cristatum*-fermented loose tea at different fermentation temperatures, and it was found that higher temperature increased antioxidant activity in the loose tea [72]. The effects of different fermentation temperatures were evaluated, and 37 °C was considered the optimum temperature to produce better ferric reducing ability, which was also favourable for increasing DPPH scavenging ability in fermented tea. However, they noticed that the increase in fermentation temperature decreased the hydroxyl radical scavenging ability in fermented tea, for which 28 °C was the optimum temperature. Thus, increasing fermentation temperature had varying effects on ferric reducing, hydroxyl radical scavenging, and DPPH scavenging in fermented tea. It is important to monitor how quickly the process will reach a pH value lower than 4.0 since this can inhibit the growth of most food-borne pathogens [73]. Some microorganisms can increase or decrease pH value. For example, at the beginning of the fermentation of caper berries, lactic acid bacteria growth rapidly lowers the pH value of the medium, displacing other microbial groups such as enterobacteria [1]. Meanwhile, the release and production of some compounds can also increase or decrease pH value.

### 5.3. Selection of Liquid Fermentation and Solid-State Fermentation

The fermentation type was also important for the fermentation effect. As is known, the vital change between solid-state fermentation (SSF) and liquid fermentation (LF) is the water content, which would affect the interactions between microorganisms, enzymes, liquids, air, and fermentation matrix. Slightly different from traditional SSF, the plant-based food substrates should supply enough nutrients such as carbon and nitrogen for microbial utilization, which put forward higher requirements for the fermentation features of strains and the control of fermentation conditions [74]. While the LF needs a larger scale fermentation vessel to provide controlled conditions such as optimum temperature, pH value, and others. It occurs in a liquid media with high water content, and all nutrients are present in the liquid medium for microbial growth, which might be a challenge for the enrichment of products. It is shown in their advantages and limitations in Table 2 for microorganism growing [75].

## 6. Conclusions

**Figure 1:**
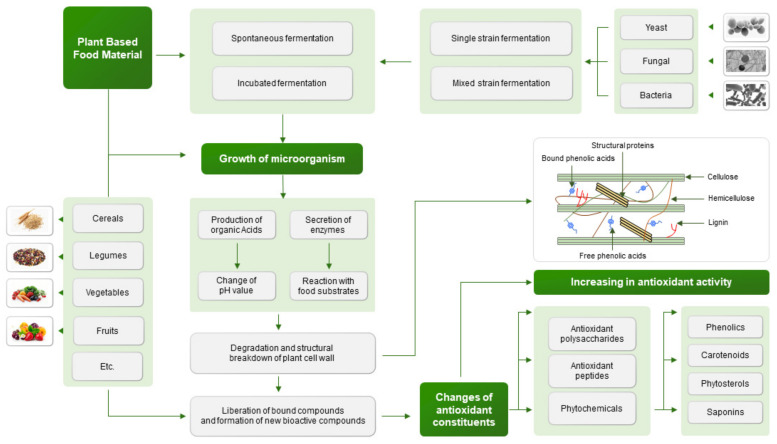
The process by which antioxidants are released and produced by fermentation.

In this work, we described microbial bioconversion in the chemical composition of various food items. Fermented food is a good source of bioactive compounds compared to most nonfermented food. Fermentation can increase amino acids and isoflavone content, and it helps to improve the ratio of nutritive to antinutritive components in plants, which encourages the production of new functional foods (Figure 1). Microorganisms such as *L. rhamnosus*, *L. plantarum, Rhizopus oligosporus*, *Aspergillus niger*, *Saccharomyces cerevisiae,* etc., lead to a series of reactions that result in higher antioxidant activity. This activity is linked to increases in antioxidant compounds, such as phenolic compounds, which have been converted from conjugated form to free form by enzymes produced by microorganisms. With the continuous development of fermentation technology, more studies are needed to discuss this topic. Fermentation’s ability to increase bioactive components, especially antioxidants in plant-based food materials, could be used to design new functional food.

Several factors influence the effect of fermentation on the liberation and production of antioxidants from plant-based food materials, including starting microorganisms, pH value, fermentation time, fermentation type, and enzymes. More studies are needed on the activities of relevant enzymes and their interactions with food during fermentation. Research has suggested that the type of food and food composition influence fermentation results. For example, it has been found that gelatinization of cereals affects the behaviour of microorganisms. Overall, fermentation can enhance the functional properties of food. This review has provided helpful information on the effect of the fermentation process on the bioactive substances and antioxidant activity of different plant-based food items.

## Figures and Tables

**Table 1:** Effect of fermentation on antioxidant activity of plant-based food material.

Food Material	The Used Microorganism	Effect on Bioactive Compounds of Plant-Based Food Material	Fermentation Type	Literature
Wheat	*Lactobacillus fermentum* (MR13), *L. rhamnosus* (C249, C1272), *L. plantarum* (LB102, LB124, LB126, LB245, 29DAN, 83DAN, 6BHI, 98A) and *L. brevis* (3BHI)	- The ferulic, p-coumaric, cinnamic, caffeic, sinapic, p-hydroxybenzoic, and gallic acids were found in the fermented dough. - *Lactobacillus plantarum* (29DAN, 83DAN) fermentation increased the carotenoid content in the Wheat dough.	Liquid and solid	[50]
Oats	*Monascus anka* GIM 3.592	- The fermented oat contained a higher content of soluble and insoluble phenolics than raw oat.	Liquid and solid	[51]
Goat milk	*Lactobacillus plantarum* 60	- The fermented milk contained antioxidative peptides such as VGINYWLAHK and DLLER.	Liquid	[52]
Buckwheat, wheat germ, barley, and rye	*Lactobacillus rhamnosus*, and *Saccharomyces cerevisiae*	- Fermentation with *L. rhamnosus* had a positive effect on DPPH inhibitory effect in some cereals but with *cerevisiae* had no significant effect on DPPH inhibitory influence in some cereals.	Liquid and solid	[15]
Defatted soybean flour	*Monascus purpureus* or *Aspergillus oryzae*	- Almost no change in the total phenolic content or antioxidant activity was found.	Solid	[28]
Bergamot juice	Pure and mixed cultures of *L. plantarum* subsp. *plantarum* PTCC 1896, *L. plantarum* AF1 and *L. plantarum* LP3	- Fermented samples contained higher radical scavenging activity than nonfermented samples. - The ternary fermented bergamot juices had a higher radical-scavenging effect than the control sample.	Liquid	[33]
Whole soybean flour	*Lactobacillus casei*	- The fermentation increased hydroxyl radical scavenging and DPPH scavenging activities.	Liquid and solid	[32]
Kidney beans	Solid-state fermentation was carried out by *Bacillus subtilis*, whilst liquid state fermentation was performed either by natural fermentation (NF) or by *Lactobacillus plantarum* strain (LPF)	- It was demonstrated that fermentation of kidney beans exhibited high soluble phenolic compound content and antioxidant activity. - It was concluded that solid-state fermentation showed more soluble phenolic content than liquid state fermentation.	Liquid and solid	[39]
Rice bran	It steamed with α-amylase, fermented with lactic acid bacteria, and hydrolyzed with complex enzymes	- Fermentation increased total phenolic content, total flavonoids, total FRAP, and ORAC values leading to higher antioxidant activity. - Twelve phenolic compounds were found after the fermentation of rice bran.	Liquid and solid	[36]
Cocoa beans	13 naturally existing yeast strains	- Yeast starter culture fermentation enhanced the total polyphenols and flavonoid content. - The *Pichia kudriavzevii* and *Hanseniaspora thailandica* fermentation were characterized with higher antioxidant potential compared with natural fermentation.	Liquid and solid	[17]
Elderberry juices	Ten strains of *Lactobacillus*	- Fermentation increased anthocyanins and flavanol glycosides and produced phenyllactic acids. - *Lactobacillus rhamnosus and Lactobacillus plantarum* induced the increase of total polyphenols.	Liquid	[14]
Sea buckthornberries	*Lactobacillus plantarum*, *Lactobacillus plantarum* subsp. *argentoratensis* and *Oenococcus oeni* strains	- It was proved that *Lactobacillus plantarum* strains are the most effective because they increase the flavonols and the antioxidant activity of sea buckthorn–apple juices.	Liquid	[30]
Guava leaves tea	It first fermented with *Monascus anka* and *Saccharomyces cerevisiae* and then hydrolyzed with complex enzymes	- It was found that fermentation increased the total flavonoids, total phenolics, quercetin, kaempferol, reducing the power of the soluble phenolics, and antioxidant activity. - Quercetin was the dominant phenolic compound among different phenolics.	Liquid and solid	[34]
Chickpeas	*Cordyceps militaris* SN-18	- Solid-state fermentation possessed higher total phenolic content and antioxidant activity.	Solid	[43]
Buckwheat flours	Selected lactic acid bacteria (LAB) and *Rhizopus oligosporus* fungi	- It was indicated that liquid-state fermentation with selected lactic acid bacteria (LAB) and *Rhizopus oligosporus* fungi could improve the antioxidant activity of buckwheat flours.	Liquid	[53]

**Table 2:** Comparison between solid-state fermentation and liquid fermentation.

Factor for Comparison	Solid-State Fermentation	Liquid Fermentation
Advantages	The medium is simple, inexpensive and easily available No more pretreatments of substrates Low contaminations Forced aeration is often easier Simplified and minimized downstream process and waste disposal Simple equipment for fermentation High volumetric productivity	Easiness of measuring process parameters Smooth distribution of nutrients and microorganisms Ability to control growth parameters and conditions Availability of high-water content for the growth of microbes
Limitations	Low moisture level may restrict the microbial growth Not removing metabolic heat in a large scale Difficulties in monitoring the process parameters	Use of expensive media and expensive equipment Complex and expensive downstream process and difficulty in the waste disposal High power consumption

## Data Availability

The data presented in this study are available are included in the article.
